# Factors Influencing Delays in Patient Access to New Medicines in Canada: A Retrospective Study of Reimbursement Processes in Public Drug Plans

**DOI:** 10.3389/fphar.2019.00196

**Published:** 2019-03-29

**Authors:** Sam Salek, Sarah Lussier Hoskyn, Jeffrey Roy Johns, Nicola Allen, Chander Sehgal

**Affiliations:** ^1^School of Life and Medical Sciences, University of Hertfordshire, Hatfield, United Kingdom; ^2^Outcome Research Division, Institute for Medicines Development, Cardiff, United Kingdom; ^3^Innovative Medicines Canada, Ottawa, ON, Canada; ^4^Global Pricing and Product Strategy, Precision Xtract, London, United Kingdom

**Keywords:** reimbursement, time to list, Canada, patient access to new medicines, CADTH, health technology assessment

## Abstract

Individuals who rely on public health payers to access new medicines can access fewer innovative medicines and must wait longer in Canada compared to major markets around the world. New medicines/indications approved by Health Canada and reviewed for eligibility for reimbursement by the Common Drug Review or the pan-Canadian Oncology Drug Review (CDR/pCODR) from the beginning of 2012 through to the end of December 2016 were analyzed, with data taken from the relevant bodies’ websites and collected by IQVIA. This analysis investigated individual review segments – Notice of Compliance (NOC) to Health Technology Assessment (HTA) submission, HTA review time, pan-Canadian Pharmaceutical Alliance (pCPA) negotiation time, and public reimbursement decision time, and analyzed the trends of each over time and contributions to overall time to listing decisions. Average overall timelines for public reimbursement after NOC were long and most of this time is taken up by HTA and pCPA processes, at 236 and 273 days, respectively. This study confirms that Canadian public reimbursement delays from 2013-2014 to 2015-2016 lengthened from NOC to listing (Quebec + 53%, first provincial listing + 38%, and country-wide listing + 22%), reaching 499, 505, and 571 days, respectively. Over the same period, time from NOC to completion of HTA has increased by 33%, and time from post-HTA to first provincial listing by 44%. The pCPA process appears to be the main contributor to this increasing time trend, and although some provinces could be listing more quickly post-pCPA, they appear to be listing fewer products. Reasons for large delays in time to listing include the many-layered sequential process of reviews conducted before public drug plans decide whether to provide access to new innovative medicines. Although there has been some headway made in certain parts of the review processes (e.g., pre-NOC HTA), total time to listing continues to increase, seemingly due to the pCPA process and other additional review processes by drug plans. More clarity in the pCPA and provincial decision-making processes and better coordination between HTA, pCPA, and provincial decision-making processes is needed to increase predictability in the processes and reduce timelines for Canadian patients and manufacturers.

## Introduction

The publicly financed health insurance system in Canada covers all Canadians for hospital and physician services for free at the point of service, and is provided through federal, and highly decentralized provincial and territorial plans (Marchildon, 2013). The Canadian health system is somewhat unique, in that prescription medicines are not covered under Medicare unless they are covered as part of a hospital-based service (as per the *Canada Health Act*); thus Canada has developed its own unique network of prescription drug coverage to meet the needs of its population. As of January 1, 2018, 35.5 million Canadians are eligible for some kind of drug coverage plan (representing 98% of the Canadian population) (Dinh and Sutherland, 2017). Only two other countries in the Organization for Economic Co-operation and Development (OECD) have such a high private insurance component of total healthcare spending: the United States and Slovenia (OECD, 2017). Canada’s spending on pharmaceuticals is comparable to the wealthiest OECD countries (ranked fifth among 31 OECD countries in 2015 for expenditure per capita on pharmaceuticals; US$685), as is its share of health care spending (pharmaceuticals represent 14.4% of total healthcare expenditure) (OECD, 2017).

The federal, provincial and territorial governments provide drug plans that cover various populations. Although around two-thirds of the population is estimated to be eligible to be covered by a public drug program across the country, based on eligibility criteria (Dinh and Sutherland, 2017), only one-third of the Canadian population receives public drug plan benefits (CIHI, 2017). The Federal Government funds programs for First Nations and Inuit people, and other targeted populations such as veterans and those under the auspices of correctional services, the Canadian Forces, and the Royal Canadian Mounted Police. Provincial and territorial public drug plans likewise cover various specific populations and each plan makes decisions about eligibility coverage criteria and benefits. The majority of Canadians who are eligible for these plans are seniors and low-income individuals and, in certain provinces, other populations that may not have private insurance – such as self-employed or working in trades with limited or no private health benefits, or youth over 18 years old that are not studying full-time. Some provinces have Pharmacare (a monopolistic government-run public plan under which everyone is eligible), however, most individuals do not actually qualify to receive any reimbursement benefits because their deductibles are set high compared to their actual drug expenses in order to protect against catastrophic drug costs (they would only pay minimal out-of-pocket expenses if they were also enrolled in a private plan that paid their deductible) (Dinh and Sutherland, 2017). Forty-three percent (43%) of prescription drug costs in Canada are paid by federal, provincial and territorial public plans for all publicly covered populations (CIHI, 2016).

Approximately 22.5 million (62% of Canadians) were covered for prescription drugs by a private insurance plan in 2015 and 36% of Canada’s prescription drugs costs are covered by private insurance (either employer-based or individual contracts) (CIHI, 2016). In most provinces, private drug plans are voluntary –although private insurance is mandatory for eligible workers in Quebec (Barnes and Anderson, 2015). The remainder of prescription drug costs (22%) are estimated to be paid out-of-pocket through deductibles or co-pays or by individuals who do not actively participate in a prescription drug plan. In fact, the Conference Board estimates that fewer than 1 million Canadians (1.8% of the population) were not eligible for any insurance in 2018, and that an additional 3.6 million Canadians (10% of the population) did not enroll in plans eligible to them in January 2018 (Dinh and Sutherland, 2017).

Health Canada is the federal government department responsible for regulating the quality, safety and efficacy of medicines in accordance with the *Food and Drugs Act*. If the medicine under review meets safety, efficacy and quality requirements, marketing authorisation may be granted and either a NOC or NOC with conditions (NOC/c) is issued. The medicine is then eligible to be reviewed for formulary listing and reimbursement by both public and private plans. Each federal, provincial and territorial public plan makes decisions about coverage and establishes their own formularies. Formularies set by private plans are generally more generous in terms of drug coverage than public plans (Rovere and Skinner, 2016). Although private plans do impose certain types of restrictions in terms of criteria for reimbursement, 80% of private plans in Canada are “open plans” without any exclusions in terms of their formularies (TELUS Health, 2017).

Public plans have extensive review processes, relying heavily on the HTA reviews conducted primarily from a public drug plan perspective. In other words, the value of medicines is evaluated in terms of health services and budgeting in the context of public drug plans, whereas private payers place greater emphasis on employee productivity, benefits packages, and recruitment tools (Stewart, 2015; Sanofi, 2017). These differences in review priorities may contribute to the fact that private plans take a quarter of the time to add medicines to their lists for reimbursement (4 months) compared to public plans (16 months) (Rovere and Skinner, 2016). However, as the utilization of medicines has increased and given that specialty drugs for serious conditions are impacting their budgets, private plans are turning to either their own economic evaluation processes or to the public drug plans’ HTA process in search of additional cost-containment measures (IQVIA, 2016).

In the past, many Canadian provincial governments conducted their own clinical and economic evaluations to inform decisions on medicine listing for their public programs. CADTH is a third-party, government-funded HTA organization, created the intergovernmental CDR in 2002. The CDR systematically assesses the comparative clinical effectiveness and the cost-effectiveness of products with new active substances and all new indications being requested for reimbursement. The purpose of these assessments is to make more efficient recommendations on specific reimbursement criteria and to enhance consistency in decision making across different public drug plans managed at the federal or provincial level for medicines provided outside of a hospital setting (CADTH, 2016), which in Canada represents about 89% of the total pharmaceutical market (IQVIA, 2016).

An alternative HTA review program was created in 2010, initially called the “joint oncology drug review,” specializing in oncology medicines used in hospital and retail settings (even oncology medicines administered in hospital and palliative care oncology medicines are reviewed). Later renamed pCODR, the program makes recommendations to public drug plans and provincial cancer agencies that make reimbursement decisions for cancer medicines, specifically. pCODR’s remit is “to bring consistency and clarity to the assessment of new cancer drugs, by looking at both clinical evidence and cost-effectiveness” (CADTH, 2018b).

In the province of Québec, INESSS conducts HTAs and makes recommendations to the MSSS accordingly (Québec, 2010).

When a manufacturer wishes a new product to be reimbursed by public plans, it must send its submission to CADTH for review by the CDR, or pCODR, or to INESSS for public listing in Québec. After receiving an HTA recommendation from the appropriate body, provincial, territorial and/or federal plans may jointly negotiate with manufacturers through the pCPA, but subsequently maintain the ability to make their own decisions about inclusion of new products in their formularies (i.e., decisions to list the product or not). Each provincial plan makes reimbursement decisions on the basis of their reviews of the cost-effectiveness and budget impact of a new medicine and has the ability to further negotiate prices with manufacturers thereafter. While public plans often align with CADTH’s recommendations, enabling provincial payers to consider their local context in their decision-making illustrates the flexibility of the process (Allen et al., 2016). In Québec, the Minister of Health makes decisions on coverage, although there are stricter rules in place that are intended to ensure that the Minister’s decisions are aligned with the recommendations of INESSS.

**FIGURE 1:**
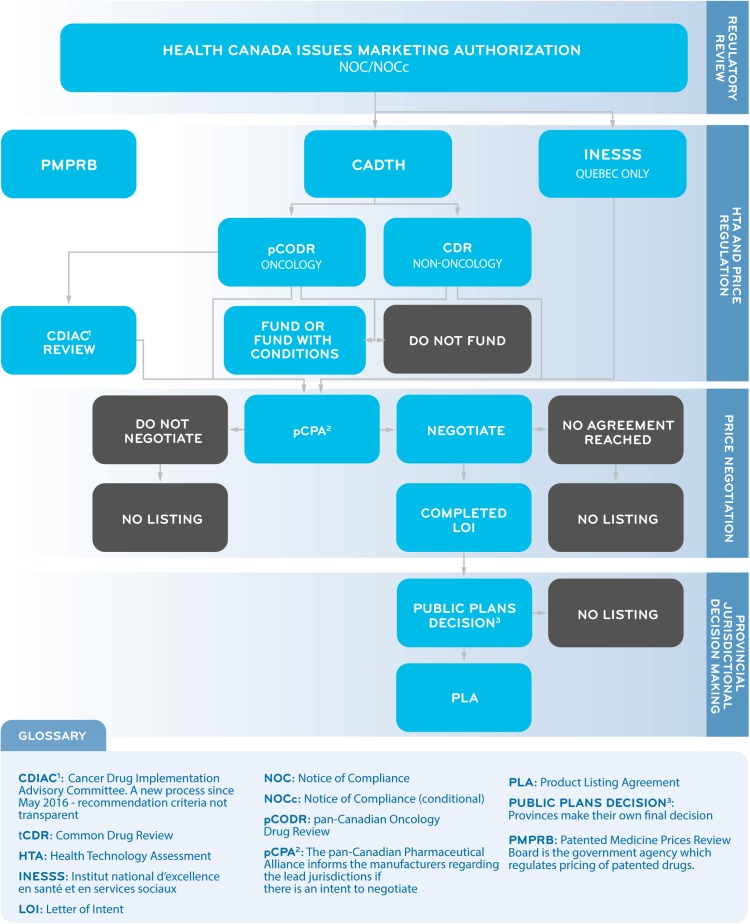
A Simplified Overview of the Public System Reimbursement Decision Pathway for New Medicines.

The pCPA was established by the Council of Federation (a collective of provincial and territorial premiers) in 2010 as a pan-Canadian body for the purpose of conducting joint public drug plan negotiations for brand and generic medicines in Canada. The goal of these negotiations is to achieve “greater value for publicly funded drug programs and patients.” If an agreement is reached, a LOI is signed by the manufacturer and the pCPA, and each participating jurisdiction will decide whether and when to fund the medicine through its own public drug plan through a confidential product-listing agreement with the manufacturer (Council of the Federation, 2014; Milliken et al., 2015). A successful pCPA negotiation does not guarantee drug listing by all participating provinces or simultaneous listings in multiple provinces. The public system reimbursement decision pathway for new medicines is summarized in Figure 1.

The drug plan managers in each province also decide which beneficiaries of their drug plan will be covered for the medicines listed on the formulary. Due to budgetary and other province-specific considerations, each of the provinces may use slightly different criteria to determine whether a medicine or indication should be listed leading to differences in listing practices from one province to another, such as the number of medicines that are listed overall, the number of new medicines that are added to the list each year, which indications are covered for each medicine and for what clinical criteria, and how much of the medicine’s cost will be paid by the province (Wong and Jepson, 2011).

Since 2012, an early-to-market approval scheme, called “pre-NOC HTA process” was introduced for both CDR and pCODR regimes. Since March 2018, CDR submissions, like pCODR submissions, can be made up to 6 months prior to NOC (CADTH, 2018a). Since 2017, INESSS has also created its own formal pre-NOC scheme consistent with CADTH (informally available since 2016 for oncology products only, and by recommendation of the Minister only), in order to better align with the timing of pCPA negotiations (INESSS, 2017).

Canadians who rely on public health payers to access medicines must wait significantly longer compared to individuals in major markets around the world (Millson et al., 2016), and Canadians who rely on private plans (Rovere and Skinner, 2016). There is a significant regulatory approval lag for some new medicines in Canada vs. United States and major European Union markets (Shajarizadeh and Hollis, 2015). Furthermore, once regulatory approval is obtained, Canadians wait longer than those in the United States and many major European markets to obtain public reimbursement for new medicines (Millson et al., 2016). Probable reasons for the larger delays include the sequential, multi-layered review processes (as described above) conducted before public drug plans decide whether to provide access to innovative medicines. For example, the time-to-listing of new medicines approved by Health Canada from 2004 to 2013 by at least one public health plan by January 2015 was substantially longer than for listing by at least one private plan (on average 468 days compared to 132 days, respectively). Furthermore, coverage for new medicines by public plans is also more limited than by private plans. Over the same period, only 50% (231 out of 464) of new medicines approved by Health Canada in 2004–2013 were listed by one or more public plans, whereas 89% (413 out of 464) were covered by at least one private plan by January 2015. Only 11 out of 231 publicly listed medicines were listed faster by public plans than private plans (Rovere and Skinner, 2016).

The aim of our research, therefore, was to develop and report a comprehensive analysis of time-to-reimbursement by public payers in Canada and to explore the contributing factors to total delays in public reimbursement. Although private payers’ total time-to-reimbursement was beyond the scope of this study, we note that there is evidence that private payer product listing agreements are generally completed within 6 months of starting the negotiation (PDCI, 2015, 2016).

## Materials and Methods

### Study Design

We conducted a review of the total time from NOC to public reimbursement broken out into individual review processes for the nine provincial jurisdictions participating in CADTH (NIHB, the only federal drug plan with available listings data, is excluded due to limited data points available since its entry into the pCPA process in early 2016). We also conducted separate analyses for certain metrics not related to HTA for Quebec because it is the only province that does not participate in the pan-Canadian CADTH processes; INESSS conducts HTAs independently. The study design for this research followed the STROBE Initiative’s recommendations for reporting observational studies (von Elm et al., 2014). The time period under review is from the beginning of 2012 through to the end of December 2016.

### Data Sources

Canadian Agency for Drugs and Technology in Health reimbursement recommendations issued through the CDR and pCODR process between 2012 and 2016 and available on CADTH website were cross-referenced with the Health Canada NOC database (Health Canada, 2017b) to confirm the NOC date for all new product submissions and indications submitted to CADTH. Information from the pCPA website was used to identify the negotiation status and outcomes up to the end of December 2016, and provincial plan websites including Quebec were used to obtain provincial listing status and dates. Funding information for cancer medicines was obtained from the pCODR Provincial Funding Summary reports. All information up to June 2016 was collected by IQVIA. Information to the end of 2016 was supplemented by the authors.

Information about the populations receiving public drug plan benefits was collected from CIHI’s National Prescription Drug Utilization Information System (NPDUIS) database upon request for the nine provincial jurisdictions, and from the Health Ministry’s annual report in the case of Quebec (RAMQ, 2017).

### Data Analyses

The measure used in our analysis is the mean or average, being careful to include volumes whenever applicable. No adjustment is made for changing volumes since much of the processes are standardized and not meant to be impacted by different volumes.

The primary analysis compared: (1) the number of medicine indications listed; and (2) the time to listing in Quebec, the time-to-first listing, and time-to-country-wide listing in the other nine jurisdictions. Given that Canada does not have a universal public drug plan covering every Canadian, the status of pan-Canadian drug listings can be complex to understand and measure. Thus, a measure unique to Canada of broad-based or near-universal listing was developed to proxy consistent listing across the country. We use the term “country-wide listing” to refer to this measure. Country-wide listing was calculated based on a new medicine or indication being listed in a number of public plans that together cumulatively represent at least 80% of the Canadian population receiving public plan benefits (excluding Quebec’s public beneficiary population; as earlier quoted, representing one/third of the Canadian population). For example, if a product was listed in Ontario (that province representing 40% of the public drug plan beneficiary population in the nation), in British Colombia (representing another 30% of the population) and in Manitoba (representing 10%), then the cumulative 80% would be considered to have achieved country-wide listing. This approach is consistent with previous approaches of comparing Canada’s reimbursement timelines with other countries that have single national drug coverage plans (Millson et al., 2016).

A medicine was considered ‘listed’ if it had either a full or restricted listing status by December 2016 – the latter including coverage under a special or exceptional access program on the formulary of a provincial drug plan or cancer agency. Time-to-listing was evaluated as the number of calendar days from the date of market authorization (NOC) to the date of public reimbursement (i.e., when the medicine or indication was listed by a jurisdiction). In this way, time-to-listing is an indicator of the time taken for public payers to review and include new medicines in their formularies and, accordingly, the time taken for Canadian patients to have access to them. The dates of marketing authorization, of CADTH submission and recommendation, as well as of public reimbursement were available for the exact day, month, and year, whereas the dates of pCPA negotiation start and end were only available for the month and year, with the last day of the month being posted by pCPA regardless of the actual day. As a result, pCPA timelines could be ±30 days. Timelines for individual review segment for the nine jurisdictions (all provinces excepting Quebec) were calculated separately, representing all relevant decision and action points by manufacturers and reviewers – from regulatory approval to HTA recommendations to pCPA negotiation outcomes and to public listing decisions.

Due to the fact that there were a different number of products for each review segment, there were two ways of calculating time to listing depending on the analysis. The first was to start from listed products only, and calculate the respective times for each review segment for that basket of products – this is a more precise and accurate measure of time to listing for the basket of products that eventually received listing; however, it does not capture the timelines of medicines that did not achieve public listing during the period under review in this study. Thus, a second method was employed to calculate the average time for each individual review segment, separately, for the respective cohort of products that went through a particular review process (e.g., average time for all products that went through CADTH; average time for all products that went through pCPA; etc.), and then adding them together. Although this is not as precise of a picture for the products that eventually obtain listing, we believe it is a just representation of the duration of each individual review process for the entire cohort of new medicines and indications that pass through the review system, including those that ultimately may not succeed at being listed.

Time to first provincial listing was evaluated as the number of calendar days between when a NOC was issued and when the medicine indication was listed by the first of any of the nine CADTH-partner provinces. Time to country-wide listing was taken as the unweighted time from NOC until listed in provinces (excluding Quebec) cumulatively covering at least 80% of the population receiving public drug plan benefits.

As a final step in the data analysis, a time series analysis of total time to listing was performed, comparing 2013-2014 and 2015-2016 timeframes. We also conducted a sub-analysis for oncology products so as to generate information specific to listing decisions in this particular field of medicine.

## Results

### Sequential Process and Effect

**FIGURE 2:**
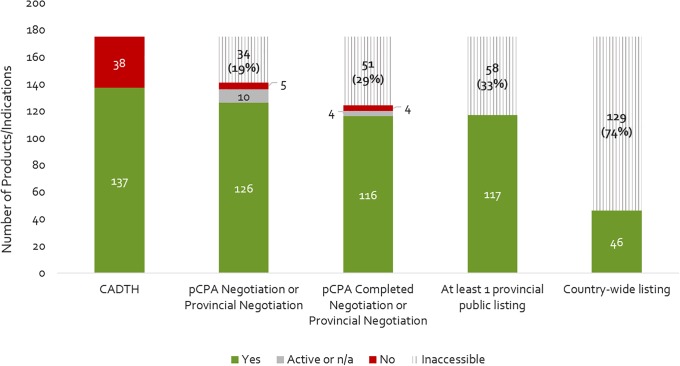
Number of products that have completed a CADTH HTA review (2012- December 2015), a pCPA negotiation, and achieved at least one provincial listing or country-wide listing as of December 2016. Yes, passed the respective review step; Active, still under review on December 31, 2016; n/a, review not yet begun; No, did not pass the respective review step; Inaccessible, not available as a reimbursed benefit to eligible beneficiaries in the plan(s) – reflects the filtering effect.

Figure 2 provides a summary of the review outcomes and listing decisions as of December 2016. Approximately 22% of products reviewed by CADTH were issued a negative recommendation, and the remainder (78%) received a positive or conditional recommendation. Almost all products reviewed and approved by CADTH were selected by the pCPA for joint negotiation or for individual provincial negotiation (10% were not – note that the pCPA also opted to negotiate on a few products that received a negative CADTH recommendation), and the large majority of these were negotiated (8% were not). Among those products having been negotiated to completion, most were listed in at least one province plus a few which had received a negative CADTH recommendation or not been successfully negotiated by pCPA. Cumulatively, 33% of the 175 new medicines and indications that were reviewed by CADTH by 2015 did not get listed in any one province. Moreover, only 26% of new products/indications reviewed by CADTH achieved country-wide listing by the end of 2016. These results indicate that the sequential process from NOC to listing filters out a large portion of new medicines.

### Sequential Time to Listing

**FIGURE 3:**
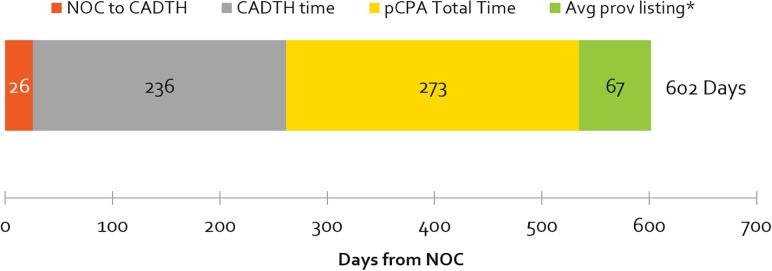
Average number of cumulative and segmental days from NOC to at least one provincial listing, by review agency, 2012–2016 (^∗^excludes products with artificial pCPA dates of completed negotiations published January 31, 2014). Average times to listing in Quebec are excluded. Note that the pCPA total time includes time from when the CADTH recommendation is issued, to the start of a pCPA negotiation. pCPA times could be ±30 days, as negotiation start and end times are published as the last day of every month.

The number of days from a product receiving marketing approval (NOC) to it being listed in any one province (excluding Quebec), by review agency, is shown in Figure 3. The average cumulative time from NOC to listing was 1.7 years (602 days). Most of this time was taken up by the HTA and pCPA processes – at 39% and 45% of total cumulative review time, respectively. Average provincial listing times are for products ranging from one to nine provincial listings.

### Public Reimbursement Timelines, Over Time

**FIGURE 4:**
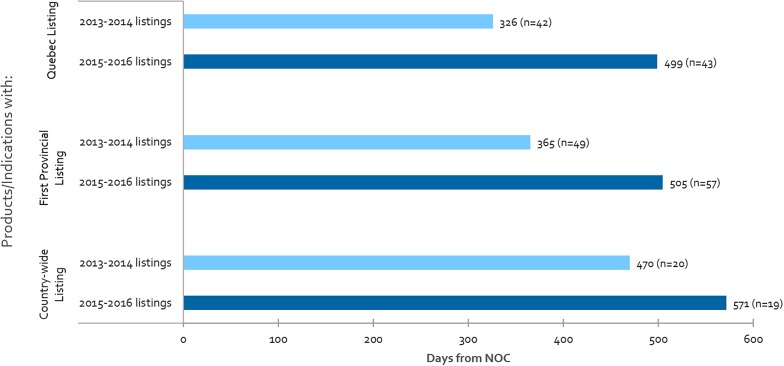
For all CADTH reviewed products, average number of days from NOC to: Quebec listing, first provincial listing in at least one province other than Quebec, and country-wide listing excluding Quebec, where “n” denotes the number of listings in each respective period.

Listing times are shown in Figure 4 for Quebec, first provincial listing and country-wide listing among other provinces, for products that achieved listing in 2013-2014 as compared to 2015-2016. Our analysis indicates that Canadian public reimbursement delays have lengthened in time from NOC to provincial listing in Quebec (53% increase), to first provincial listing other than Quebec (38% increase), and to country-wide listing excluding Quebec (21% increase). Although there was a simultaneous increase in the volume of products and indications (16%) for our first provincial listing measure, no increase was observed in Quebec nor for our country-wide listing measure.

### Contribution to Timeline Before and Up to the End of HTA vs. Post-HTA Processes (Including pCPA and Public Plan Decisions)

**FIGURE 5:**
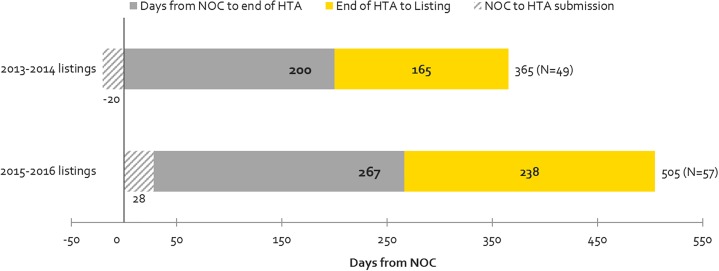
Time to First Provincial Listing, in number of days, (all provinces, excluding Quebec), as segmented based on the following timelines: from NOC to HTA submission; from NOC to HTA recommendation; and from HTA recommendation to listing (source: CADTH reviews, excluding resubmissions).

Figure 5 shows the time taken for review processes up to the HTA decision (upstream) and post-HTA (downstream) for the same products examined under time-to-first provincial listing in Figure 4, above. Downstream times have increased from 2013-2014 to 2015-2016 to a greater extent than upstream (44 vs. 33% increase in number of days) but the volume of products listed has also increased. The volume increase may be related to the CDR backlog experienced in 2013-2014, when CDR began experiencing resourcing issues which delayed the start of many reviews that manufacturers submitted in 2013 and 2014. This pushed many listings that would have occurred in 2013-2014 to the 2015-2016 period. The backlog could also explain the 33% or 67-day increase in the time taken from NOC to HTA recommendation for products listed in 2015-2016 vs. 2013-2014. Post-HTA timelines increased by 44% or 73 days, and this can likely be explained in part by slower pCPA negotiation start times and negotiation durations in 2016 (Millson, 2017).

### Oncology vs. Non-oncology Public Reimbursement Timelines – Recent Trends

**FIGURE 6:**
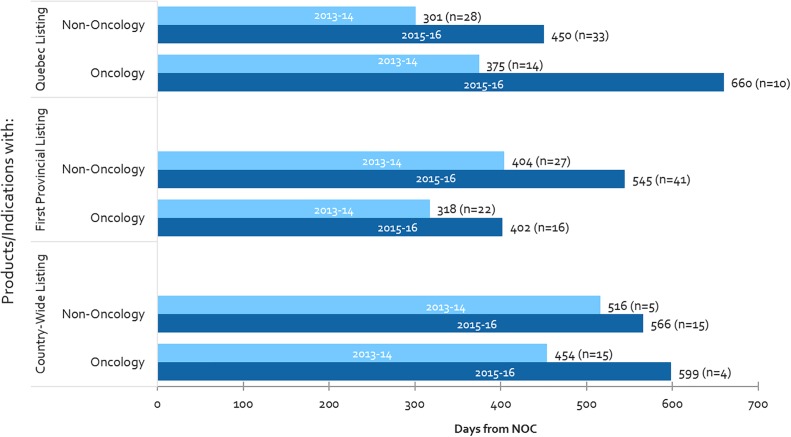
Public reimbursement timelines for oncology vs. non-oncology products, and number of items listed (n), 2013-2014 and 2015-2016.

From 2013-2014 to 2015-2016, oncology listing timelines have increased in all three measures in Canada (Quebec, first provincial listing and country-wide listing), with the largest increase being observed in Quebec (76% or 285 days increase), followed by country-wide listing (32% or 145 days increase) (Figure 6). With respect to the time-to-first provincial listing, despite the 26% increase in oncology-specific timelines, the time taken to list oncology products still remains shorter than for non-oncology products (by 143 days in 2015-2016). Oncology timelines in 2015-2016 had surpassed non-oncology product timelines in both Quebec and country-wide (oncology timelines were already longer than non-oncology in Quebec in 2013-2014).

Moreover, whereas oncology listing timelines have increased, the number of oncology products being listed have fallen – as a result, one can presume that volumes are not related to the increasing timelines observed for oncology products. On the other hand, both timelines and the number of non-oncology listings have increased, thus could be related, for example through the CDR back-log as mentioned above. The decline in the number of oncology listings could be the result of the longer timelines to list oncology products, creating a backlog of products still undergoing review.

### Time to First Provincial Listing, NOC to End of HTA vs. Post-HTA – Oncology vs. Non-oncology

Supplementary Figure S1 shows the time to first provincial listing for the same cohort of products presented in Figure 6 but in this case the timeframes for listing are separated into the sequential processes upstream and downstream. For non-oncology products, the source of overall increases in listing times stem from both upstream (NOC to HTA recommendation) and downstream (post-HTA) processes (34% increase in time each or 79 and 60 days, respectively). The increased timeline from NOC to HTA recommendation is likely due to the CDR backlog as mentioned above, whereas the post-HTA timeline increases are likely due to slower pCPA negotiation start times and durations mentioned earlier. For oncology products, however, the source of increase is entirely the post-HTA timeframe (59% increase in time, or 90 days). In contrast to that for non-oncology products, the NOC to HTA recommendation timeline decreased slightly – likely due to increased frequency and/or earlier pre-NOC submissions (see next section). Moreover, the increase in post-HTA timelines was greater for oncology products than for non-oncology products (59% or 90 days vs. 34% or 60 days, respectively). These trends are observed despite a lower volume of oncology products being listed in 2015-2016 vs. 2013-2014.

### HTA Review Timelines for CDR, pCODR and INESSS

**FIGURE 7:**
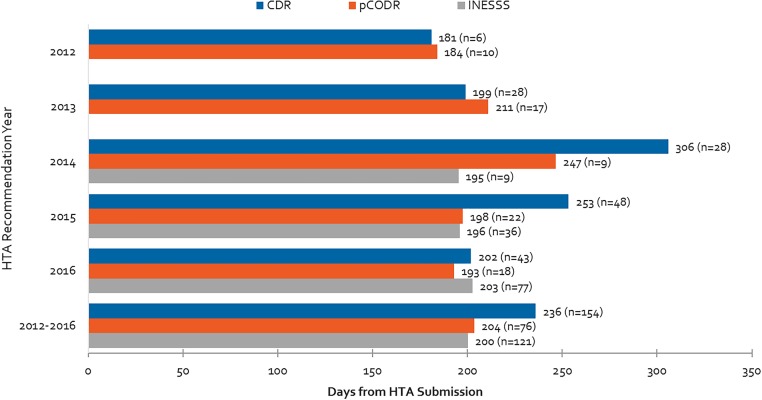
Health Technology Assessment review times for CDR, pCODR and INESSS and number of manufacturer submissions from 2012 to 2016. Data for INESSS represents products with completed recommendations by INESSS that had Health Canada NOCs starting in January 2014. As a result, very few reviews in 2014 are captured (likely starting in October 2014).

Average review times for different HTA bodies are presented in Figure 7 for the 2012 to 2016 period. The aforementioned backlog for the CDR process can be observed starting in 2013 and peaking in 2014. This backlog was resolved after the relevant agencies began to impose application fees (Health Canada, 2017a). Although in 2016 HTA reviews were generally meeting the agencies’ own target timelines of 180 days, on average the CDR exceeded its target timelines over the 2012–2016 period due to the backlog in 2013–2015. Although INESSS was generally in line with target CDR and pCODR timelines in 2014 and 2015, 2016 trends appear to indicate that review timelines are lengthening. This may or may not be due to increasing volumes of reviews processed in 2016.

### Differences in Time From NOC to First Provincial Reimbursement Between Submissions Made Pre-NOC vs. Post-NOC

**FIGURE 8:**
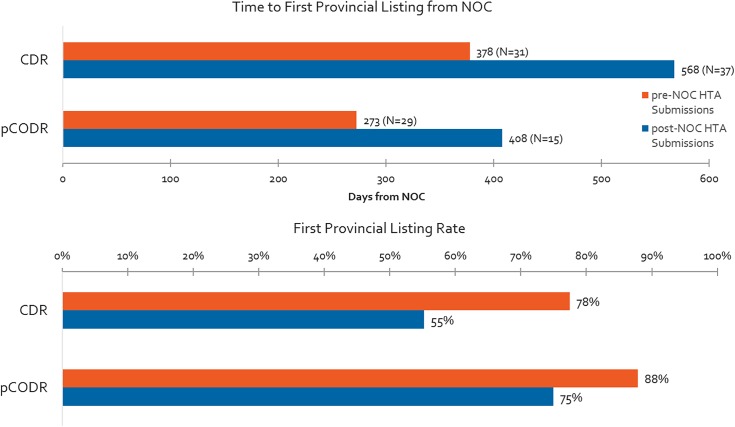
Time to first provincial listing from NOC for products having pre- vs. post-NOC HTA submission, and % listing rates for pre- and post-NOC HTA manufacturer submissions (products with CADTH recommendation to December 2015, time to first provincial listing to December 2016).

Time to first provincial listing is shown in Figure 8 for products with pre-NOC HTA submissions, and products with post-NOC HTA submissions. On average, non-oncology products with pre-NOC HTA submissions (CDR) are listed approximately 6 months (190 days) earlier than those having submitted post-NOC. Similarly, for oncology products, submissions (pCODR) made pre-NOC are listed approximately 4 months (135 days) earlier than products with submissions made post-NOC. Likewise, non-oncology products with pre-NOC HTA submissions have a 42% greater probability of achieving listing in at least one province (excluding Quebec) as compared to those with post-NOC HTA submissions, while oncology products with pre-NOC HTA submissions experience a 17% greater probability of being listed as compared to those with post-NOC HTA submissions.

### Pre-NOC HTA Submission Trends for CDR and pCODR – Frequency and Timelines

**FIGURE 9:**
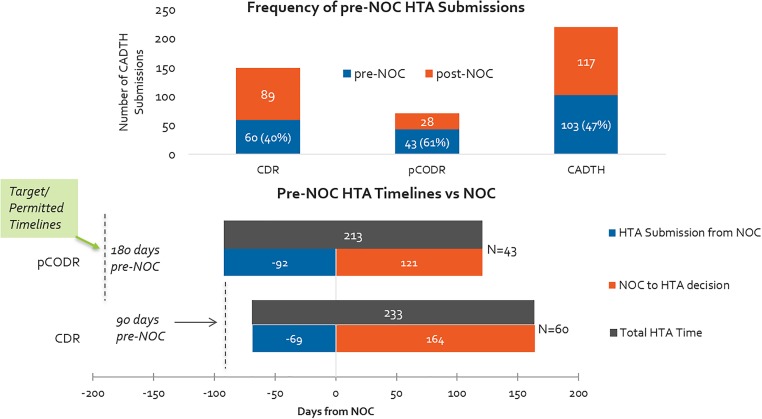
(Top) Percentage of HTA submissions submitted pre- and post-NOC, 2012–2016 (CADTH only). (Bottom) HTA submission timelines with respect to target/permissible timelines for pre-NOC filing, pCODR vs. CDR.

Overall, oncology products are submitted on the basis of parallel review processes (pre- and post-NOC submissions) more often than non-oncology products. More than 60% of pCODR submissions included in our study period were submitted pre-NOC, compared to 40% of CDR submissions (Figure 9). Although submissions were permitted 6 months ahead of the expected NOC date for oncology products, in practice the actual timelines for submissions was half of that (3 months before NOC). Submissions for non-oncology products were permissible 3 months in advance of expected NOC, and on average applicants submitted two-thirds of the way into this prescribed period. INESSS did not have a pre-NOC submission process during our study period.

### Relative Time to First Provincial Listing Between HTA Substitutions Made Pre-NOC vs. Post-NOC – Trend Over Time

Time to first provincial listing between 2013-2014 and 2015-2016 are shown in Supplementary Figure S2, as presented in terms of whether products had pre-NOC HTA submissions or post-NOC HTA submissions. For products listed in 2013-2014, non-oncology products with parallel HTA reviews were 75% faster (3.5 months) in achieving first provincial listing, while oncology products were listed 78% faster (2 months) under the same circumstances. However, by 2015-2016, the gap widened significantly in time to achieve first provincial listing between products with pre-NOC and post-NOC HTA submissions.

In 2015-2016, oncology products with pre-NOC HTA submissions experienced a 28% increase (68 days) in time to first provincial listing as compared to the 2013-2014 time period (despite a lower volume), while oncology products reviewed post-NOC experienced a 115% increase (359 days) in the time taken to achieve first provincial listing in the 2015-2016 vs. 2013-2014 periods. As a result, in 2015-2016, the gap has widened fourfold to reach 358 days longer (12 months), for a product with post-NOC HTA review, to reach first provincial listing, compared to pre-NOC HTA.

Non-oncology products with pre-NOC HTA reviews experienced a 25% increase (84 days) in time-to-listing in 2015-2016 compared to 2013-2014, while those undergoing post-NOC HTA reviews saw a 50% increase (223 days) in time to first provincial listing over the same time period. Accordingly, in 2015-2016, the gap in time to first provincial listing increased by more than twofold to reach 251 days longer (8.4 months) for non-oncology products reviewed post-NOC vs pre-NOC.

Whereas oncology products with post-NOC HTA submissions had shorter time to first provincial listing than non-oncology products in 2013-2014 (129 days shorter), in 2015-2016 they had slightly longer time to first provincial listing than non-oncology products with post-NOC HTA submissions, and their volume had dropped significantly, whereas that of non-oncology products had increased.

## Discussion

Currently, although there are performance target timelines set for Health Canada’s and CADTH’s reviews, there are no such targets set for the following review steps including the pCPA process i.e., how soon a negotiation is initiated and its duration, and public plans’ decisions to list after negotiations are completed. The pCPA process can take a few weeks to more than a year in some cases (PCPA, 2017), and public plans can take as long as they want to, given budget realities. The development of a formal pCPA process provides an opportunity to introduce target time frames while taking into account the need for both timely decisions and sufficient time for manufacturers and public drug plan administrators to negotiate efficiently. Similar target timeframes have been developed to manage the review processes in other jurisdictions for regulators, HTA agencies, and public plans alike (European Union, 1988).

This study confirms that Canadian public reimbursement delays have lengthened in time from NOC to listing, and that post-HTA times are the biggest contributor to the increasing delays. This is especially notable for oncology review timelines, which in some cases, surpass non-oncology product timelines, resulting in a decline in the number of oncology medicines being listed. The CDR backlog that began in 2013 was resolved starting in 2015 following the implementation of application fees that must accompany submissions and in 2016 the HTA review process was generally meeting its target timelines of 180 days. On the other hand, recent evidence suggests that pCPA negotiation timelines continued to be lengthy in 2017 compared to 2016 (Millson, 2017).

Parallel processes like pre-NOC submissions represent an opportunity both in terms of time gained and probability of listing. Over our entire study period, parallel processes, such as those allowing for pre-NOC submissions, reduced timelines by an average of 6 months to reach first provincial listing for non-oncology products and by 4 months for oncology products, and pre-NOC submissions had a 42% and 17% greater probability of achieving listing in the respective cases of non-oncology and oncology submissions. Also, note that pre-NOC submission criteria at the beginning of our study period were more limited for the CDR process compared to the pCODR process, likely explaining the difference observed in frequency. The CDR pre-NOC process was initially reserved for products granted Priority Review by Health Canada. However, although manufacturers utilize the parallel review process more frequently for oncology vs. non-oncology products, pre-NOC HTA submissions were still under-utilized in general terms. In particular, manufacturers making submissions for oncology products tended to submit within the final three of the 6 months available in advance of the expected NOC date, while manufacturers of non-oncology products tended to wait two out of the 3-month pre-NOC period before submitting. It is unknown what impact, if any, the recent process change by CDR to allow up to 6 months before NOC (from 3 months only) to make a submission for non-oncology products will have on accelerating the HTA submission timelines, and ultimately, the overall time to list.

The advantage observed seems to have been mainly driven from the widened gap observed in 2015-2016, which was not the case in the earlier period. In 2013-2014, there was minimal advantage of utilizing parallel reviews in terms of lessening time to listing (an approximately 2-3-month reduction in time). However, the gap widened in 2015-2016 between pre-NOC and post-NOC HTA submissions resulting in a delay of 8 months to a year for post-NOC submissions as compared to pre-NOC submissions. This gap between pre- and post-NOC timelines seems to indicate longer post-HTA timelines specifically for products that undergo the standard, post-NOC HTA submission process. Additionally, whereas there used to be an advantage for oncology products submitted post-NOC vs. non-oncology post-NOC, it appears that is no longer the case. Moreover, time to first provincial listing has also increased for pre-NOC HTA submissions, bringing into question the potential for parallel reviews to reduce overall time to list.

Before the creation of the CDR process in 2002, each of the Canadian provinces had their own review boards, but conducting multiple reviews was a costly and an inefficient use of resources (Spitz, 2013). Furthermore, not all provinces had the capacity to conduct full HTAs. Although the creation of new incremental and sequential processes, such as the CDR in 2002, pCODR in 2011, and the pCPA in 2010 to replace multiple and uneven provincial negotiations, may have reduced the workload for the provinces, the timeliness and effectiveness of the reimbursement system have not improved in recent years.

This study found that the mean time was 273 days for pCPA to start and complete negotiations of CADTH-reviewed products between 2012 and 2015, with an additional 67 days to reach at least one provincial listing (up to December 2016) (Figure 3). This combined total of 340 days is very similar to findings from a study by Milliken et al. (Milliken et al., 2015) that reported mean times to list of 324 days in nine jurisdictions following CDR or pCODR recommendation between September 2007 and August 2010, i.e., before the establishment of the pCPA. They also reported mean times to list of 279 days following CADTH listing recommendation between September 2010 and August 2013 for products that completed a pCPA negotiation, i.e., after pCPA was established. With a timeline of 340 days from our study over the 2012–2016 period, this comparison reveals that the timeline situation does not appear to have improved since the last major study assessing the impact of pCPA on timelines – and in fact, appears to have worsened, since our study indicated an increase of 44% in average post-HTA timelines to list in 2015-2016 compared to 2013-2014. Accordingly, it can be concluded that delays in patient access to new medicines have grown and continue to grow.

Thanks to the availability of pre-NOC submissions, manufacturers have succeeded in shortening the gap between NOC and HTA submissions, and CADTH has made headway in reducing the overall time to HTA recommendation from NOC. However, total time to listing and patient access from the time of regulatory approval continues to increase. The pCPA process appears to be the main contributor to this increasing time trend. More coordination and alignment between the HTA and pCPA processes, and between pCPA and provinces, could increase predictability in processes and timelines for industry.

### Limitations

There are some limitations of this study that should be stated. Analysis of the situation with regard to Quebec and individual review components including INESSS was limited due to shorter time period for data points collected. pCPA negotiation data is incomplete. For example, it is lacking precise dates which could add or subtract 30 days in pCPA calculations and is lacking some negotiation start dates and LOI dates, as well as precise indications negotiated, and participating jurisdictions including lead jurisdictions. This study also did not consider timelines for negative listing decisions by public plans due to limited and inconsistent data in our study period for non-oncology products, specifically. This study also did not include listing timelines for medicines that fall outside of publicly available formularies, e.g., hospital non-oncology medicines, some rare disease medicines paid for by other budgets (non-drug plan), etc.

## Conclusion

A number of implications emerge from this study:

• The sequential review processes inherent to the Canadian public reimbursement process make decisions time-consuming and burdensome, resulting in long delays for Canadians who rely on public plans for their needed medicines.
• The lack of performance targets in some processes are making the process less predictable both for industry as well as for Canadian patients. Likewise, the lack of end to end performance targets, e.g., from NOC to listing, over and above individual agency accountability, could be hampering the successes of individual agencies that are meeting their own performance targets.
• The lack of coordination between different review processes is increasing the delays in access to new medicines for Canadian patients across the country.
• Parallel reviews applied across the entire reimbursement process have the potential to improve time to listing and overall efficiency, benefitting all stakeholders. Better coordination amongst different review processes can be explored using the HTA and regulatory parallel processes as an example (pre-NOC). Opportunities exist to add predictability and transparency in the process between HTA and pCPA and between the pCPA and the provincial listing processes.
• For example, having objective end to end performance targets would further lend support to parallel reviews to reduce overall timelines.

More research needs to be conducted to identify opportunities to shorten overall time from NOC to provincial listing. The source of delays being experienced during the pCPA process and the extent to which increasing post-HTA timelines are due to the conduct or policies of the provinces themselves needs further exploration. Further study should look at opportunities to take an integrated and system view perspective and build on the strengths and successes of individual processes, to reduce system-level inefficiencies and to explore parallel reviews and other mechanisms to improve overall timelines for Canadian patients’ access to new medicines.

## Author Contributions

SS contributed to study design, data analysis, interpretation of the results, and first draft of the manuscript. SLH contributed to study design, data collection, analysis, interpretation of the results and manuscript review. JJ contributed to interpretation of the results and first draft of the manuscript. NA reviewed the several drafts of the manuscript. CS contributed to study design, interpretation of the results and review of the manuscript.

## Conflict of Interest Statement

SLH and CS were employed by company Innovative Medicines Canada, Canada. NA was employed by company Precision Xtract, United Kingdom. The remaining authors declare that the research was conducted in the absence of any commercial or financial relationships that could be construed as a potential conflict of interest.

## Supplementary Material

Figure S1 Time to first provincial listing for non-oncology (CDR) and oncology (pCODR) product HTA submissions made pre-NOC vs. post-NOC, 2013–2014 and 2015–2016.

Figure S2 Time to First Provincial Listing, in number of days, (all provinces, excluding Quebec), for oncology and non-oncology products, as segmented based on the following timelines: from NOC to HTA submission; from NOC to HTA recommendation; and from HTA recommendation to listing (source: CADTH reviews, excluding resubmissions).

## Abbreviations

CADTH: Canadian Agency for Drugs and Technology in Health
CDIAC: Cancer Drug Implementation Advisory Committee
CDR: Common Drug Review
CEDAC: Canadian Expert Drug Advisory Committee
HTA: Health Technology Assessment
INESSS: Institut national d’excellence en santé et en services sociaux (National Institute for Excellence in Health and Social Services)
LOI: Letter of Intent
MSSS: Ministère de la Santé et des Services sociaux (Minister of Health and Social Services)
NOC: Notice of Compliance
NOCc: Notice of Compliance with Conditions
pCODR: Pan-Canadian Oncology Drug Review
pCPA: Pan-Canadian Pharmaceutical Alliance
PMPRB: Patented Medicine Prices Review Board
