# Supplementary figures and images for: Factors Influencing Delays in Patient Access to New Medicines in Canada: A Retrospective Study of Reimbursement Processes in Public Drug Plans
Source: Front Pharmacol. 2019 Mar 29;10:196. doi: 10.3389/fphar.2019.00196 (PMC6449480; doi:10.3389/fphar.2019.00196)

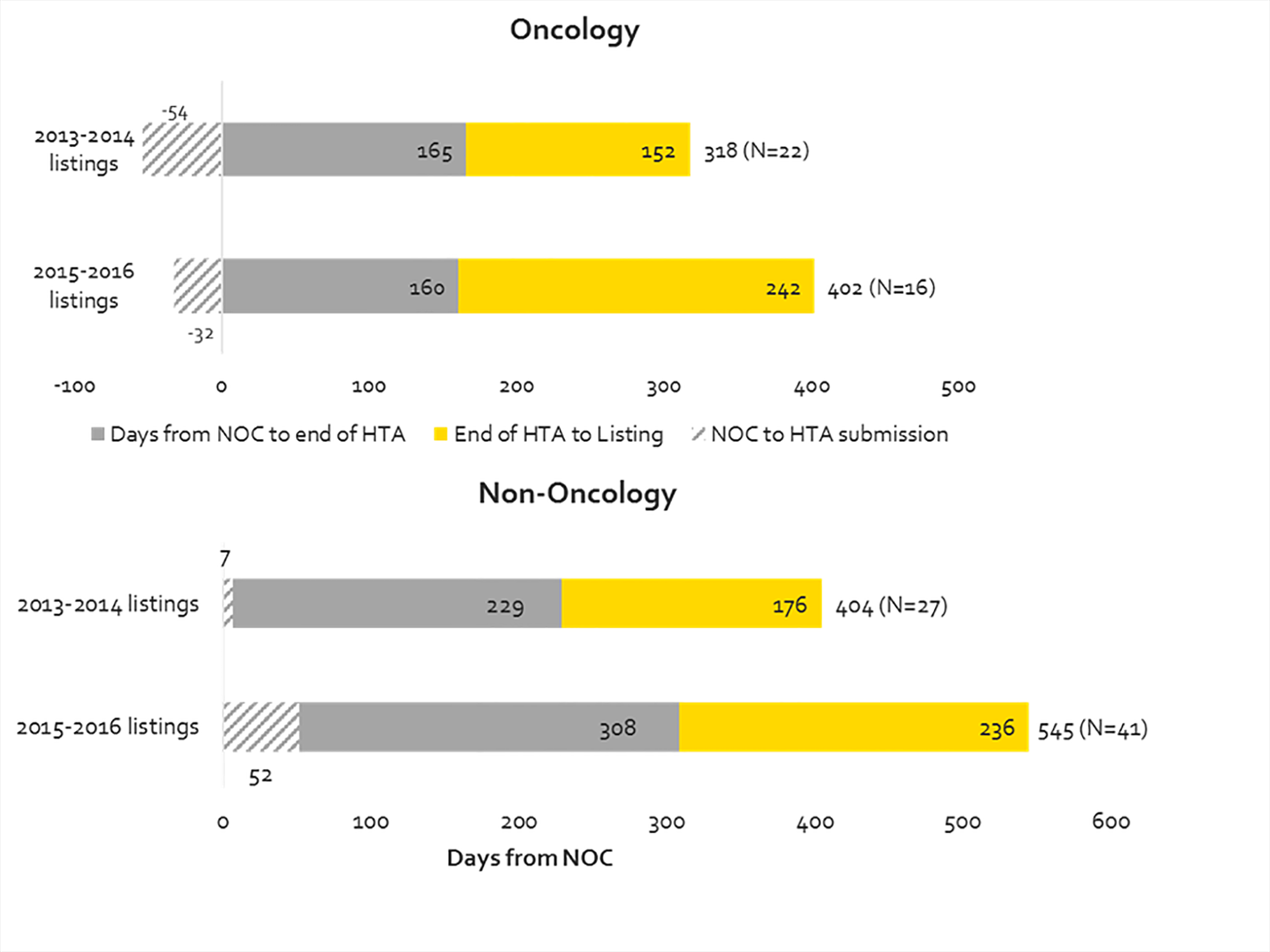

Supplement: Figure S1 — Time to first provincial listing for non-oncology (CDR) and oncology (pCODR) product HTA submissions made pre-NOC vs. post-NOC, 2013–2014 and 2015–2016. [file Image_1.TIF]

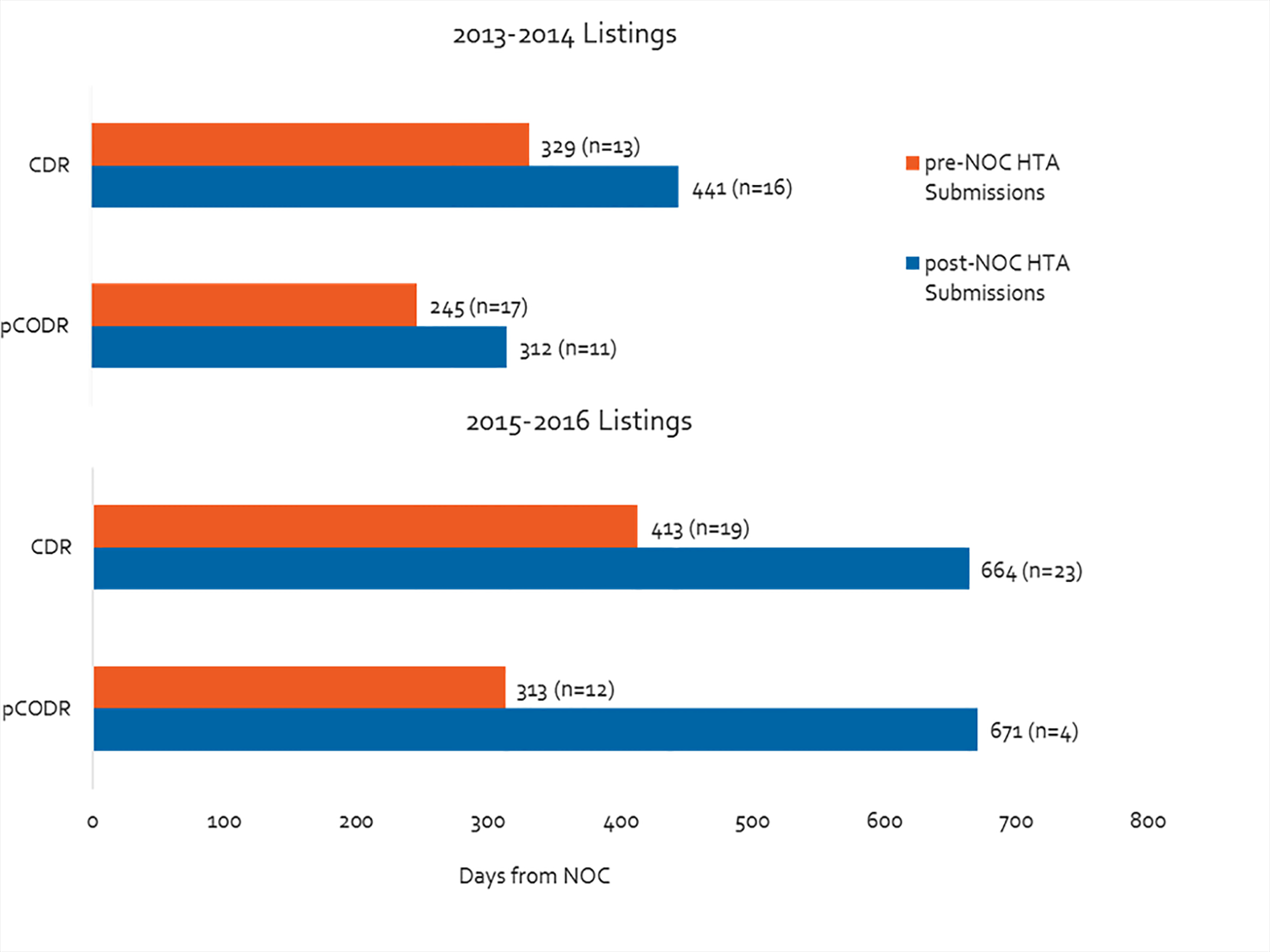

Supplement: Figure S2 — Time to First Provincial Listing, in number of days, (all provinces, excluding Quebec), for oncology and non-oncology products, as segmented based on the following timelines: from NOC to HTA submission; from NOC to HTA recommendation; and from HTA recommendation to listing (source: CADTH reviews, excluding resubmissions). [file Image_2.TIF]
